# A Case of Extensive Tuberculosis With Bacterial Infection Treated in a Peripheral Hospital

**DOI:** 10.7759/cureus.57067

**Published:** 2024-03-27

**Authors:** Maxim Suleac, Ana Rezende, Socrates Naranjo, Malam Djassi

**Affiliations:** 1 Internal Medicine, Unidade Local de Saúde do Norte Alentejano (ULSNA) Hospital Doutor José Maria Grande, Portalegre, PRT

**Keywords:** dyspnea, infectious disease, weight loss, cough, tuberculosis

## Abstract

Since 1997, the World Health Organization (WHO) publishes the Global Tuberculosis Report every year, which contains the most up-to-date and accurate statistical data on tuberculosis (TB) worldwide. TB is an infectious disease that is one of the leading causes of human morbidity. It ranks among the top 10 most common causes of death worldwide and is more likely than other pathogens, such as the human immunodeficiency virus (HIV) and acquired immunodeficiency syndrome (AIDS), to result in a fatal outcome. After the introduction of the TB vaccine, the situation improved significantly, but the disease was not defeated. In this context, we present the clinical case of a male patient who was admitted to the Emergency Department (ED) due to a productive cough, dyspnea, and weight loss. After a complementary study, he was diagnosed with extensive diffuse pulmonary TB with a bacterial infection.

## Introduction

Tuberculosis (TB) is a chronic, progressive mycobacterial infection, often with an asymptomatic latent period after initial infection. The lung is the most commonly affected organ, but it can often damage other organs and systems. The main symptoms include cough, fever, weight loss, and general malaise. The outdated name for pulmonary TB is phthisis. Until the twentieth century, TB was practically incurable. Currently, a comprehensive program has been developed to identify and treat the disease in the early stages of its development. In Portugal, the incidence of cases has been consistently decreasing, with a low incidence since 2015 (<20/100,000 inhabitants). Its concentration in large urban centers and its association with different comorbidities require joint action between health institutions and social structures [1].

## Case presentation

We present a clinical case of a 46-year-old male patient of Ukrainian origin. He is a bricklayer who has been residing in Portugal for about 15 years. He is a long-time smoker (abstinent for one month), a moderate alcohol consumer, has no chronic medication regimen, complied with the SARS-CoV2 vaccination, and was treated empirically with amoxicillin/clavulanic acid two weeks before admission due to the cough and fever. The patient was admitted to the ED due to unexplained fatigue, dyspnea, cough with colorless, odorless sputum without hemoptysis, and significant unintentional weight loss of around 20 kg in two months, which indicates progressive deterioration. The entrance examination revealed no significant changes other than tachycardia and low peripheral saturation. Analytically, there was an increase in inflammatory parameters (Table 1).

**Table 1:** Laboratory tests

Parameters	Normal	Parameter at admission	Parameter at 24 h
Leukocytosis	4-6 x 10^3^/uL	15.95 x 10^3^/μL	18.06 x 10^3^/μL
Neutrophils	1.5-8 x 10^3^/μL	13.53 x 10^3^/μL	16.13 x 10^3^/μL
C-reactive protein	0-5 mg/dL	206.3 mg/L	247.6 mg/L
Procalcitonin	0-0.5 ng/mL	1.3 ng/mL	1.9 ng/mL

Blood gas analysis showed pH of 7.46, pO_2_ 63 mmHg, pCO_2_ 26 mmHg, HCO_3_ 21.7 mmol/l, lactate 2.6 mmol/l, oxygen saturation 93.4%, with alveolar-arterial gradient G(A-a) of 12.16 mmHg. To exclude a viral lung infection, we required screening for SARS-CoV2, respiratory syncytial virus, and influenza A+B, all of which had negative results. Blood culture tests were also collected, and the results were negative. Chest x-ray demonstrated diffuse bilateral miliary hypotransparency, more extensive on the right. After suspecting TB, an acid-fast bacilli (AFB) test was performed, and the results were positive (10-99 bacilli per field). Additionally, a culture test was required, and the results were positive. Due to the primary diagnosis of TB, blood tests for HIV and hepatic viral markers were added. We requested chest computed tomographic angiography (CTA) for better clarification of the alterations found on the chest x-ray to exclude pulmonary thromboembolism and to confirm/rule out bacterial infection.

The CTA revealed a miliary pattern of diffuse, extensive, micronodular vascular interstitium with areas of multifocal consolidation, sometimes confluent. The air bronchogram was bilateral and diffuse, with a slight predominance on the right side. Three areas of thick-walled cavitation in the right apex, reaching 7 cm, were observed, suggesting extensive TB. In addition, a slight diffuse pleural thickening, with minimal effusion at the right base and multiple mediastinal adenopathies (pre-tracheal and hilar), was described. The new findings required obtaining additional patient information to identify potential risk factors for the disease and assess any possible contacts. The patient was flagged on the National Epidemiological Surveillance System (SINAVE) platform, and screening of close contacts was performed. The patient was admitted to the Internal Medicine Department under the Infectious Diseases Department collaboration, where he was treated with levofloxacin isoniazid, pyrazinamide, rifampicin, and ethambutol for pulmonary TB with bacterial infection. The patient exhibited good clinical, analytical, and some radiological evolution during the treatment.

## Discussion

**Figure 1:**
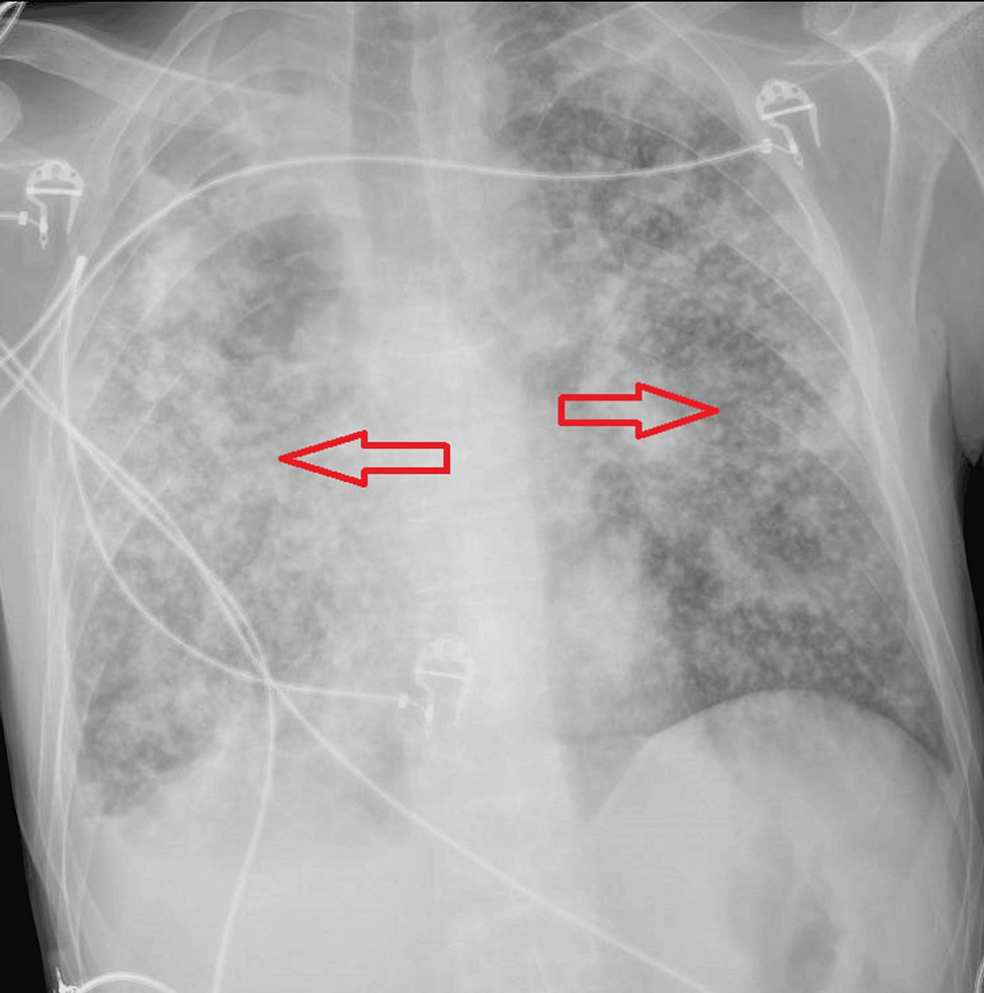
Diffuse tuberculosis miliary pattern on x-ray (marked with red arrows)

**Figure 2:**
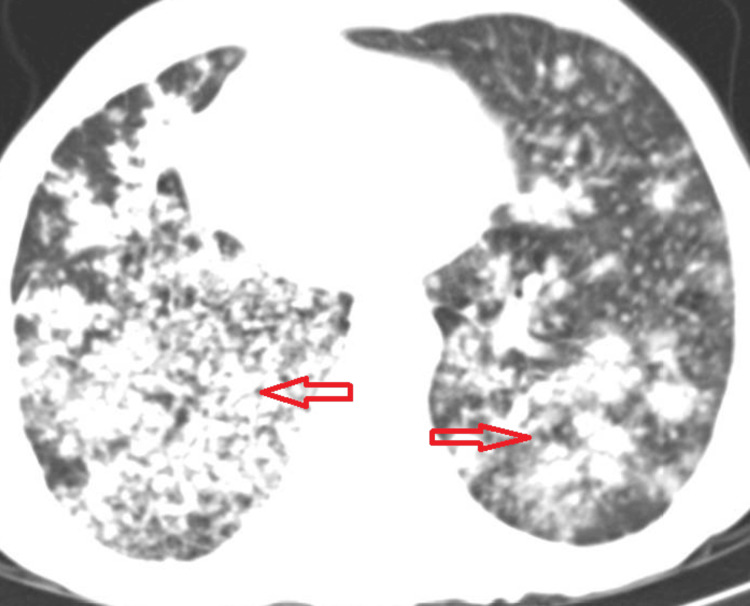
Diffuse tuberculosis miliary pattern on CT (marked with red arrows)

**Figure 3:**
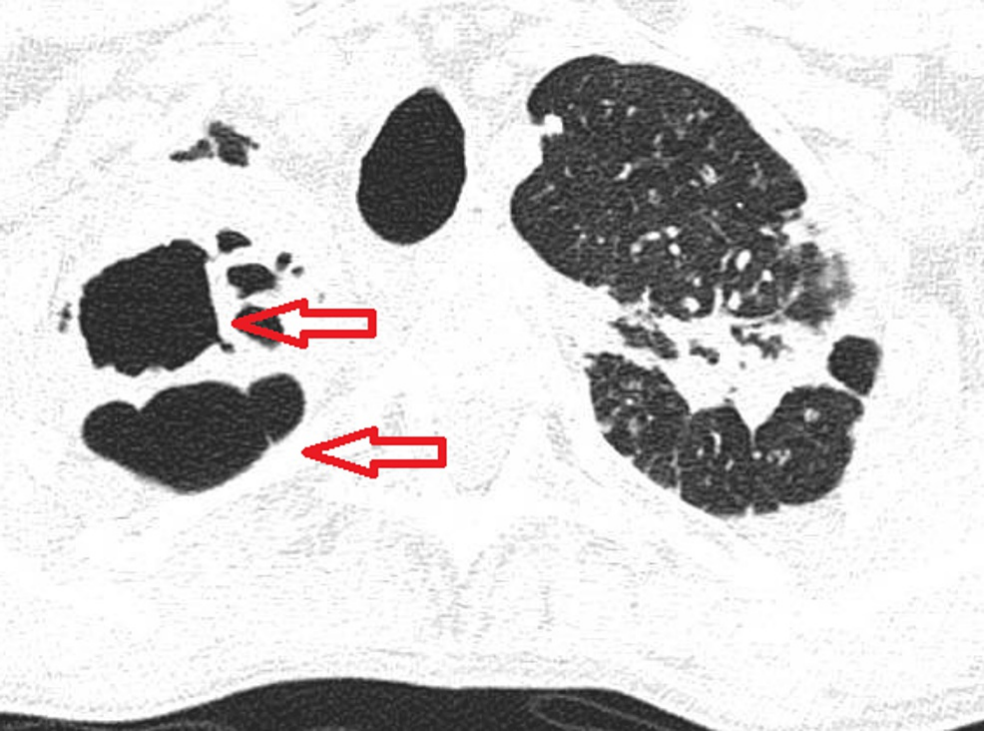
Tuberculosis cavitation on chest CT (marked with red arrows)

**Figure 4:**
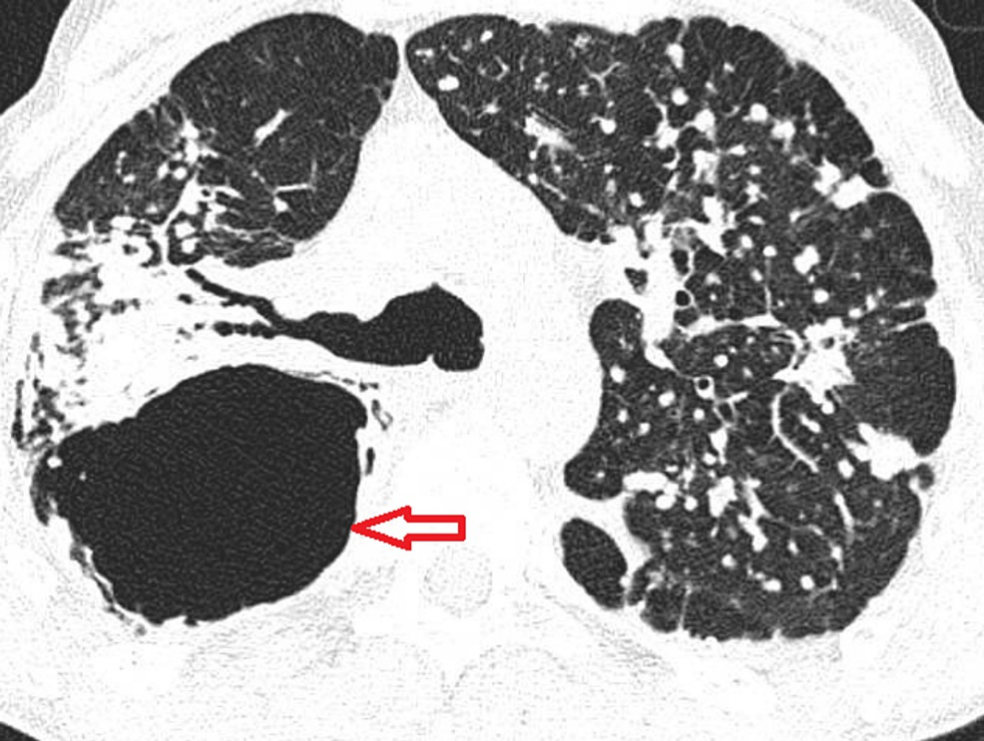
Tuberculosis cavitation observed on chest CT (marked with red arrow)

The diagnosis of TB is confirmed if the *Mycobacterium tuberculosis* (Mt) complex is identified in a culture test or if the direct examination and nucleic acid amplification test (NAAT) are positive [2]. In the face of a negative result and clinical suspicion of TB, one should insist on collecting more samples or even moving toward invasive diagnostic techniques, such as fiberoptic bronchoscopy [3]. However, the decision to initiate the empirical treatment is sometimes based on a presumptive diagnosis while taking into consideration clinical picture or imaging changes, particularly when demonstrating granulomas and/or caseous necrosis. In our case, we had a positive direct exam with pathognomonic imaging changes (Figures 1-4).

Molecular resistance tests are routinely recommended for all positive samples and are necessary for individuals with risk factors for multidrug-resistant TB [4]. Regardless of molecular tests being carried out, phenotypic tests, cultural isolation of Mt, and subsequent antibiograms are mandatory and allow for the confirmation of both the diagnosis and the viability of the bacilli present in the biological sample. In our case, a bacteriological test was also required and was subsequently positive with the isolation of *M. tuberculosis *without evidence of resistance. Regarding SARS-CoV2 infection, there is no biological reason to suspect that inactivated vaccines affect the results of the tuberculin skin test (TST) or the interferon-gamma release assay (IGRA). However, unless the TB test is considered urgent, the National Tuberculosis Controllers Association (NTCA) 2021 guidance recommends delaying testing until four weeks after the administration of the last dose of the coronavirus disease 2019 (COVID-19) vaccine [5]. Treatment is carried out with combined therapy lasting at least six months.

Our patient has completed 64 days of treatment in the hospital and was later discharged with instructions to continue treatment in an outpatient clinic, with reassessment during infectious disease consultations. Patients with a history of non-compliance receive a once-daily dose in a directly observed treatment (DOT) regimen, but this was not applied in our case. For better prevention and epidemiological control, in May 2023, the United States Preventive Services Task Force (USPSTF) recommended infection screening for all adults at increased risk of TB. This includes individuals from countries with a high prevalence of TB and individuals in shelters or correctional facilities [6]. The 2023 recommendation is based on evidence review demonstrating that available screening tools for TB (TST and IGRA) are moderately sensitive and highly specific and that treatment with available regimens confers moderate net benefit [7]. The differential diagnosis for pulmonary TB includes different causes of chronic infection, inflammatory diseases, and malignancy.

## Conclusions

Despite the remarkable progress made in controlling disease and the subsequent steady downward trend in incidence, TB, including multidrug-resistant TB, continues to pose a public health threat in most countries and should not be underestimated. TB is a preventable and treatable disease. The prognosis depends largely on the stage, location of the disease, resistance of the pathogen to drugs, and the opportunity to start the treatment. If treatment is initiated promptly, it allows for complete restoration of working capacity but does not guarantee that the disease won't relapse. Late diagnosis or inaccurate treatment can lead to significant negative health outcomes, often leading to death. The control of TB in the population is mainly achieved through early diagnosis and adequate treatment of cases, contact tracing, and the implementation of prophylaxis measures. Due to the mass migration that is taking place nowadays, a new program should be developed to identify and prevent TB in the local population, especially in the more vulnerable and low-income areas and disadvantaged families. This should include the introduction of mandatory medical examinations for migrants, especially those at risk, with further obligatory routine medical control. Also, based on the positive experience of effectively combating TB in the past, it is worth considering a mandatory vaccination of all newborns, regardless of the area of residence and risk factors.
